# Supplementary material for: Clinical characteristic and outcomes of pregnant women with COVID‐19: The PROUDEST prospective cohort study
Source: PLoS One. 2025 Jul 3;20(7):e0327174. doi: 10.1371/journal.pone.0327174 (PMC12225883; doi:10.1371/journal.pone.0327174)
Supplement: S1 Table — (DOCX) [file pone.0327174.s001.docx]

S1 Table. COVID-19 symptoms according to the pregnancy trimester of SARS-CoV-2 infection diagnosis and COVID-19 severity.

| **Symptoms** | **Total**  **n (%)** | **Pregnancy trimester of**  **SARS-CoV-2 infection diagnosis***  n (%) | | | | **COVID-19 severity****  n (%) | | | |
| --- | --- | --- | --- | --- | --- | --- | --- | --- | --- |
|  | **All**  n=260 (100.0%) | **1^st^** n=78 (30.0%) | **2^nd^** n=105 (40.4%) | **3^rd^** n=77 (29.6%) | **p** | **Non-severe** n=224 (86.2%) | **Severe** n=28 (10.7%) | **Critical** n=8 (3.1%) | **p** |
| **Fever** |  |  |  |  |  |  |  |  |  |
| No | 144 (55.4) | 37 (47.4) | 55 (52.4) | 51 (66.2) | 0.0519 | 129 (57.6) | 15 (53.6) | 0 (0.0) | 0.0046 |
| Yes | 116 (44.6) | 41 (52.6) | 50 (47.6) | 26 (33.8) |  | 95 (42.4) | 13 (46.4) | 8 (100.0) |  |
| **Cough** |  |  |  |  |  |  |  |  |  |
| No | 138 (53.1) | 48 (61.5) | 47 (44.8) | 43 (55.8) | 0.0674 | 125 (55.8) | 11 (39.3) | 2 (25.0) | 0.0710 |
| Yes | 122 (46.9) | 30 (38.6) | 58 (55.2) | 34 (44.2) |  | 99 (44.2) | 17 (60.7) | 6 (75,0) |  |
| **Dyspnea** |  |  |  |  |  |  |  |  |  |
| No | 165 (63.5) | 56 (71.8) | 63 (60.0) | 46 (59.7) | 0.1884 | 157 (70.1) | 6 (21.4) | 2 (25.0) | <0.0001 |
| Yes | 95 (36.5) | 22 (28.2) | 42 (40.0) | 31 (40.3) |  | 67 (29.9) | 22 (78.6) | 6 (75.0) |  |
| **Sore throat** |  |  |  |  |  |  |  |  |  |
| No | 186 (71.5) | 57 (73.1) | 74 (70.5) | 55 (71.4) | 0.9281 | 160 (71.4) | 18 (64.3) | 8 (100.0) | 0.1417 |
| Yes | 74 (28.5) | 21 (26.9) | 31 (29.5) | 22 (28.6) |  | 64 (28.6) | 10 (35.7) | 0 (0.0) |  |
| **Myalgia** |  |  |  |  |  |  |  |  |  |
| No | 108 (41.5) | 27 (34.6) | 32 (30.5) | 49 (63.6) | <0.0001 | 93 (41.5) | 10 (35.7) | 5 (62.5) | 0.4159 |
| Yes | 152 (58.5) | 51 (65.4) | 73 (69.5) | 28 (36.4) |  | 131 (58.6) | 18 (64.3) | 3 (37.5) |  |
| **Asthenia** |  |  |  |  |  |  |  |  |  |
| No | 192 (73.9) | 62 (79.5) | 63 (60.0) | 66 (85.7) | 0.0003 | 166 (74.1) | 18 (64.3) | 7 (87.5) | 0.4734 |
| Yes | 68 (26.1) | 16 (20.5) | 42 (40.0) | 11 (14.3) |  | 58 (25.9) | 10 (35.7) | 1 (12.5) |  |
| **Nasal congestion/discharge** | |  |  |  |  |  |  |  |  |
| No | 100 (38.5) | 25 (32.1) | 33 (31.4) | 42 (54.5) | 0.0025 | 81 (36.2) | 13 (46.4) | 6 (75.0) | 0.0504 |
| Yes | 160 (61.5) | 53 (67.9) | 72 (68.6) | 35 (45.5) |  | 143 (64.8) | 15 (53.6) | 2 (25.0) |  |
| **Diarrhea** |  |  |  |  |  |  |  |  |  |
| No | 203 (78.1) | 57 (73.1) | 80 (76.2) | 66 (85.7) | 0.1366 | 179 (79.9) | 19 (67.9) | 5 (62.5) | 0.1937 |
| Yes | 57 (21.9) | 21 (26.9) | 25 (23.8) | 11 (14.3) |  | 45 (20.1) | 9 (32.1) | 3 (37.5) |  |
| **Ageusia** |  |  |  |  |  |  |  |  |  |
| No | 108 (41.5) | 34 (43.6) | 33 (31.4) | 41 (53.2) | 0.0117 | 86 (38.4) | 18 (64.3) | 4 (50.0) | 0.0303 |
| Yes | 152 (58.5) | 44 (56.4) | 72 (68.6) | 36 (46.7) |  | 138 (61.6) | 10 (35.7) | 4 (50.0) |  |
| **Anosmia** |  |  |  |  |  |  |  |  |  |
| No | 92 (35.4) | 24 (30.8) | 26 (24.8) | 42 (54.5) | <0.0001 | 73 (32.6) | 14 (50.0) | 4 (50.0) | 0.1317 |
| Yes | 168 (64.6) | 54 (69.2) | 79 (75.2) | 35 (45.5) |  | 151 (67.4) | 14 (50.0) | 4 (50.0) |  |
| **Headache** |  |  |  |  |  |  |  |  |  |
| No | 102 (39.2) | 26 (33.3) | 27 (25.7) | 49 (63.6) | <0.0001 | 81 (36.2) | 14 (50.0) | 7 (87.5) | 0.0053 |
| Yes | 158 (60.8) | 52 (66.7) | 78 (74.3) | 28 (36.4) |  | 143 (63.8) | 14 (50.0) | 1 (12.5) |  |
| **Nausea** |  |  |  |  |  |  |  |  |  |
| No | 243 (93.5) | 70 (89.7) | 96 (91.4) | 77 (100.0) | 0.0196 | 208 (92.9) | 27 (96.4) | 8 (100.0) | 0.6280 |
| Yes | 17 (6.5) | 8 (10.3) | 9 (8.6) | 0 (0.0) |  | 16 (7.1) | 1 (3.6) | 0 (0.0) |  |
| **Vomiting** |  |  |  |  |  |  |  |  |  |
| No | 243 (93.5) | 73 (93.6) | 98 (93.3) | 72 (93.5) | 0.9974 | 215 (96.0) | 20 (71.4) | 8 (100.0) | 0.0009 |
| Yes | 17 (6.5) | 5 (6.4) | 7 (6.7) | 5 (6.5) |  | 9 (4.0) | 8 (28.6) | 0 (0.0) |  |
| **Dizziness** |  |  |  |  |  |  |  |  |  |
| No | 245 (94.2) | 75 (96.1) | 95 (90.5) | 75 (97.4) | 0.0975 | 210 (93.7) | 27 (96.4) | 8 (100,0) | 0.8210 |
| Yes | 15 (5.8) | 3 (3.9) | 10 (9.5) | 2 (2.6) |  | 14 (6.2) | 1 (3.6) | 0 (0.0) |  |
| **Skin disease** |  |  |  |  |  |  |  |  |  |
| No | 251 (96.5) | 76 (97.4) | 102 (97.1) | 73 (94.8) | 0.6319 | 217 (96.9) | 27 (96.4) | 7 (87.5) | 0.2978 |
| Yes | 9 (3.5) | 2 (2.6) | 3 (2.9) | 4 (5.2) |  | 7 (3.1) | 1 (3.6) | 1 (12.5) |  |
| **Joint pain** |  |  |  |  |  |  |  |  |  |
| No | 249 (95.8) | 77 (98.7) | 95 (90.5) | 77 (100.0) | 0.0015 | 213 (95.1) | 28 (100.0) | 8 (100.0) | 0.4606 |
| Yes | 11 (4.2) | 1 (1.3) | 10 (9.5) | 0 (0.0) |  | 11 (4.9) | 0 (0.0) | 0 (0.0) |  |
| **Other symptoms** |  |  |  |  |  |  |  |  |  |
| No | 249 (95.8) | 67 (85.9) | 84 (80.0) | 60 (77.9 | 0.4138 | 183 (81.7) | 20 (71.4) | 8 (100.0) | 0.1626 |
| Yes | 11 (4.2) | 11 (14.1) | 21 (20.0) | 17 (22.1) |  | 41 (18.3) | 8 (28.6) | 0 (0.0) |  |

* 1^st^ = SARS-CoV-2 infection diagnosis at first pregnancy trimester (4 to 13 weeks and 6 days of gestation); 2^nd^ = SARS-CoV-2 infection diagnosis at second pregnancy trimester (14 weeks to 27 weeks and 6 days of gestation); 3^rd^ = SARS-CoV-2 infection diagnosis at third pregnancy trimester (28 to 41 weeks and 6 days of gestation). ** COVID-19 severity according to the World Health Organization classification [12]. Comparative analysis was carried out by Chi-square/Fisher tests. Significance was considered at p-values >0.05.
